# Teaching potential of interdisciplinary meetings on osteoarticular infections for orthopaedic residents: Insights from a French reference center (CRIOAC)

**DOI:** 10.3205/zma001711

**Published:** 2024-11-15

**Authors:** Ramy Samargandi, Osamah Abualross, Marion Lacasse, Louis-Romée Le Nail, Julien Berhouet

**Affiliations:** 1 Orthopedic Surgery Department, Centre Hospitalier Régional Universitaire (CHRU) de Tours, Tours, France; 2 Orthopedic Surgery Department, College of Medicine, University of Jeddah, Jeddah, Saudi Arabia; 3 College of Medicine, University of Jeddah, Jeddah, Saudi Arabia; 4 Service de Médecine Interne – Maladies Infectieuses, Centre Hospitalier Régional Universitaire (CHRU) de Tours, Tours, France

**Keywords:** education, teaching methods, orthopedic, infections, communication, multidisciplinary

## Abstract

**Objective:**

Management of osteoarticular infections (OAI) represents a major public health challenge. To deal with this, the French Ministry of Health created the *Complex Osteoarticular Infections Referral Centers* (CRIOAC) in 2008. CRIOAC functions as a national network of reference centers for OAI, with the aim of coordinating, providing expertise, offering training, and conducting research at the regional level. Through multidisciplinary team meetings (MTM-CRIOAC), experts discuss complex OAI cases and formulate optimal treatment decisions. This study aims to evaluate the perception of the teaching potential of MTM-CRIOAC among orthopedic residents and identify potential areas for improvement.

**Methods:**

To conduct this cross-sectional study a validated questionnaire was distributed to orthopedic residents who participated in said meetings to evaluate their perceptions and benefit. Opinions were then collected using a Likert scale for further evaluation.

**Results:**

Results showed that (75%) found MTM useful for training, and (71%) considered it appropriate for teaching. The majority (84%) reported acquiring valuable skills, with (78%) emphasizing knowledge about complex osteoarticular infections. Residents recommended integrating more classes, active engagement, and thorough case summaries for improvement. Notably, 94% recommended MTM participation for colleagues. The study highlights MTMs’ potential in medical education, enhancing clinical skills and collaborative healthcare practices among orthopedic residents.

**Conclusion:**

Previous studies have shown that MTM-CRIOACs have improved patient outcomes. However, the literature has not yet addressed the potential teaching opportunities that MTM-CRIOACs could provide to residents. The study shows that these meetings offer grounds for teaching, enrichment of clinical judgment, professionalism, and collaborative skills of orthopedic residents.

## Zusammenfassung

**Zielsetzung:**

Das Management von osteoartikulären Infektionen (OAI) stellt eine bedeutende Herausforderung für die öffentliche Gesundheit dar. Um diese zu bewältigen, hat das französische Gesundheitsministerium im Jahr 2008 die *Centres de Référence des Infections Ostéo-articulaires Complexes* (CRIOAC) ins Leben gerufen. CRIOAC fungiert als nationales Netzwerk von Referenzzentren für OAI mit dem Ziel, die Koordination, Expertise, Ausbildung und Forschung auf regionaler Ebene zu fördern. In den interdisziplinären Teambesprechungen (MTM-CRIOAC) diskutieren Experten komplexe OAI-Fälle und formulieren optimale Therapieentscheidungen. Diese Studie zielt darauf ab, das didaktische Potenzial der MTM-CRIOAC von orthopädischen Assistenzärzten einschätzen zu lassen und potenzielle Verbesserungsmöglichkeiten zu identifizieren.

**Methoden:**

Zur Durchführung dieser Querschnittsstudie wurde ein validierter Fragebogen an orthopädische Assistenzärzte ausgegeben, die an den oben genannten Besprechungen teilnahmen, um Wahrnehmungen und Nutzen zu beurteilen. Die Meinungen wurden anschließend mit einer Likert-Skala erfasst und weiter analysiert.

**Ergebnisse:**

Die Ergebnisse zeigten, dass 75% der Teilnehmer die MTM als nützlich für die Ausbildung und 71% als geeignet für den Unterricht empfanden. Die Mehrheit (84%) berichtete, wertvolle Fähigkeiten erworben zu haben, wobei 78% das Wissen über komplexe osteoartikuläre Infektionen betonten. Die Assistenzärzte empfahlen, zur Verbesserung mehr Unterrichtseinheiten, aktive Beteiligung und ausführliche Fallzusammenfassungen zu integrieren. Bemerkenswert ist, dass 94% die Teilnahme an den MTM ihren Kollegen empfahlen. Die Studie unterstreicht das Potenzial der MTM in der medizinischen Ausbildung zur Verbesserung der klinischen Fähigkeiten und der kooperativen Gesundheitsversorgung unter orthopädischen Assistenzärzten.

**Schlussfolgerung:**

Frühere Studien haben gezeigt, dass die MTM-CRIOACs die Behandlungsergebnisse der Patienten verbessert haben. In der Literatur wurde jedoch bisher nicht das didaktische Potenzial adressiert, das MTM-CRIOACs Assistenzärzten bieten könnten. Die Studie zeigt, dass diese Besprechungen eine Grundlage für Lehre, Bereicherung der klinischen Urteilskraft, Professionalität und kooperative Fähigkeiten der orthopädischen Assistenzärzte schaffen.

## Introduction

Bone and joint infections (BJI) pose significant challenges for orthopedic surgeons in their day-to-day practice and the economic impact of treating them is substantial [1], [2]. These infections have a profound impact on the quality of life of those affected [3], [4], underlining the need to minimize their occurrence and ensure optimal care for patients suffering with such conditions. The adequate and high-quality management of BJIs represents a major challenge in public health. 

In response to this urgent issue, the French Ministry of Health took a proactive step by establishing the Complex Osteoarticular Infections Referral Centers (CRIOAC) network in 2008 [5]. These centers form a national network of reference medical centers for BJI, with a central mission to coordinate, provide expertise, offer training, and conduct research at the regional level [6]. Through this extensive network, CRIOAC assumes a pivotal role in handling the most intricate BJI cases, aiming to provide patients with the best therapeutic strategies available.

At its core, CRIOAC is committed to ensuring comprehensive and continuous care for BJI patients, from their initial hospitalization to their eventual transition to home care. By collaboration among a diverse range of healthcare professionals and leveraging their expertise, CRIOAC significantly enhances the management and overall outcomes of complex BJI cases, contributing substantially to the betterment of public health in this crucial domain [5].

Central to CRIOAC’s approach are the multidisciplinary team meetings (MTM). Each CRIOAC hosts an MTM that brings together infectious disease specialists, orthopedic surgeons, microbiologists, radiologists, medical pharmacists, and medical secretarial staff. These meetings occur on a weekly basis and discuss approximately 800 to 1000 cases annually and around 15-20 cases per week [7]. During these meetings, a wide range of BJI pathologies, including prosthetic joint and hardware infections, complex trauma cases with bone non-union, septic arthritis, osteomyelitis, and spine infections, are thoroughly presented and discussed among the assembled experts [8]. The primary goal is to arrive at the most appropriate and effective treatment decisions for each patient [2], [9].

Previous studies have shown that the MTM-CRIOAC improved patients’ outcome [5], [7]. Furthermore, as shown in a previous study by Le Nail and Samargandi conducted on tumor board meetings, they found that such meetings harbor the potential to serve as invaluable learning opportunities for orthopedic residents, by active engagement with complex cases and experts in the field. By participating in case discussions, residents gain unique insights and knowledge that can significantly enrich their education and training [10]. Despite the evident advantages observed in tumor boards the literature has not yet addressed the potential educational benefits that MTM-CRIOAC could provide for residents. Therefore, our study aims to assess the effectiveness of teaching during MTMs and identify areas for improvement. 

## Materials and methods

This survey-based study evaluates the perception of the teaching potential of a CROIAC MTM among orthopedic residents currently undergoing their orthopedic training program, or those who completed their training in the university hospital center between 2014 and 2023. Informed consent was obtained from all participants prior to completing and submitting the survey. There were 32 participants in the evaluation. The survey was not sent to residents partaking in external rotations primarily from outside centers as they were excluded from the study pool. 

For the questionnaire, validated through previous research use and modified accordingly, we built upon our experience from our previous study. Which was conducted in a similar manner, to evaluate the teaching potential of multidisciplinary tumor board meetings for orthopedic residents. Where results were positive and showed promise regarding the benefit residents received from such meetings, which compelled us to replicate it the current study [10]. For face validity, a group of three experts with significant expertise in medical education was convened. These experts were provided with access to the questionnaire and requested to assess its content, structure, and language to guarantee that the items were unambiguous, relevant, and easily understood by the intended participants. The feedback and suggestions were integrated into the final version of the questionnaire, thereby confirming its face validity.

The questionnaire was distributed to all orthopedic residents via email, which included an internet link directing them to an online form questionnaire (Google docs; Google Inc., Mountain View, California, USA). Some residents, who had completed their orthopedic residency and left the center during the study period, were considered as the senior group. The survey solely focused on various aspects of the MTM and did not cover other related elements such as the osteoarticular infection outpatient department clinic, surgeries, rounds, or weekly teaching. The questionnaire comprised both general and specific questions, and participants provided their opinions using a Likert scale and free text sections for some questions. All data collected were anonymized, and the results were compiled in an Excel spreadsheet for analysis (Microsoft Corporation, Richmond, Virginia, USA). For statistical analysis, median and range were used for continuous variables, while number and percentage (%) were employed for categorical variables. Surveys with incomplete data were excluded from the analysis.

## Results

**Figure 1:**
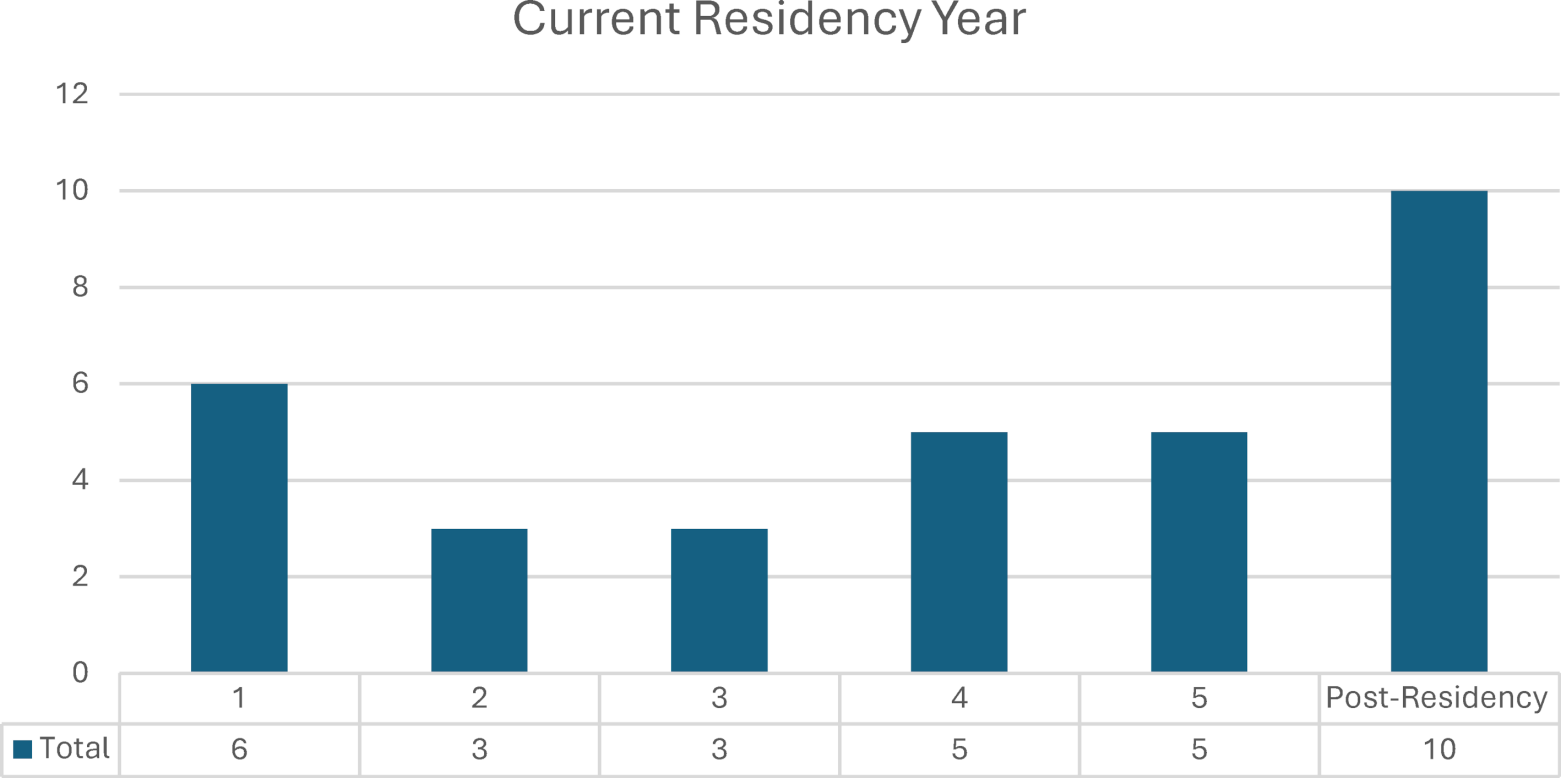
Demographic data of participants

The median year for the residents was 4^th^, with a range spanning from the 1^st^ to the 5^th^ years, as well as post-residency participants. Of the total number, there were 8 female (25%) and 24 male (75%) participants. The distribution of residents based on their advancement in the residency program is illustrated in figure 1 (Fig. 1).

**Figure 2:**
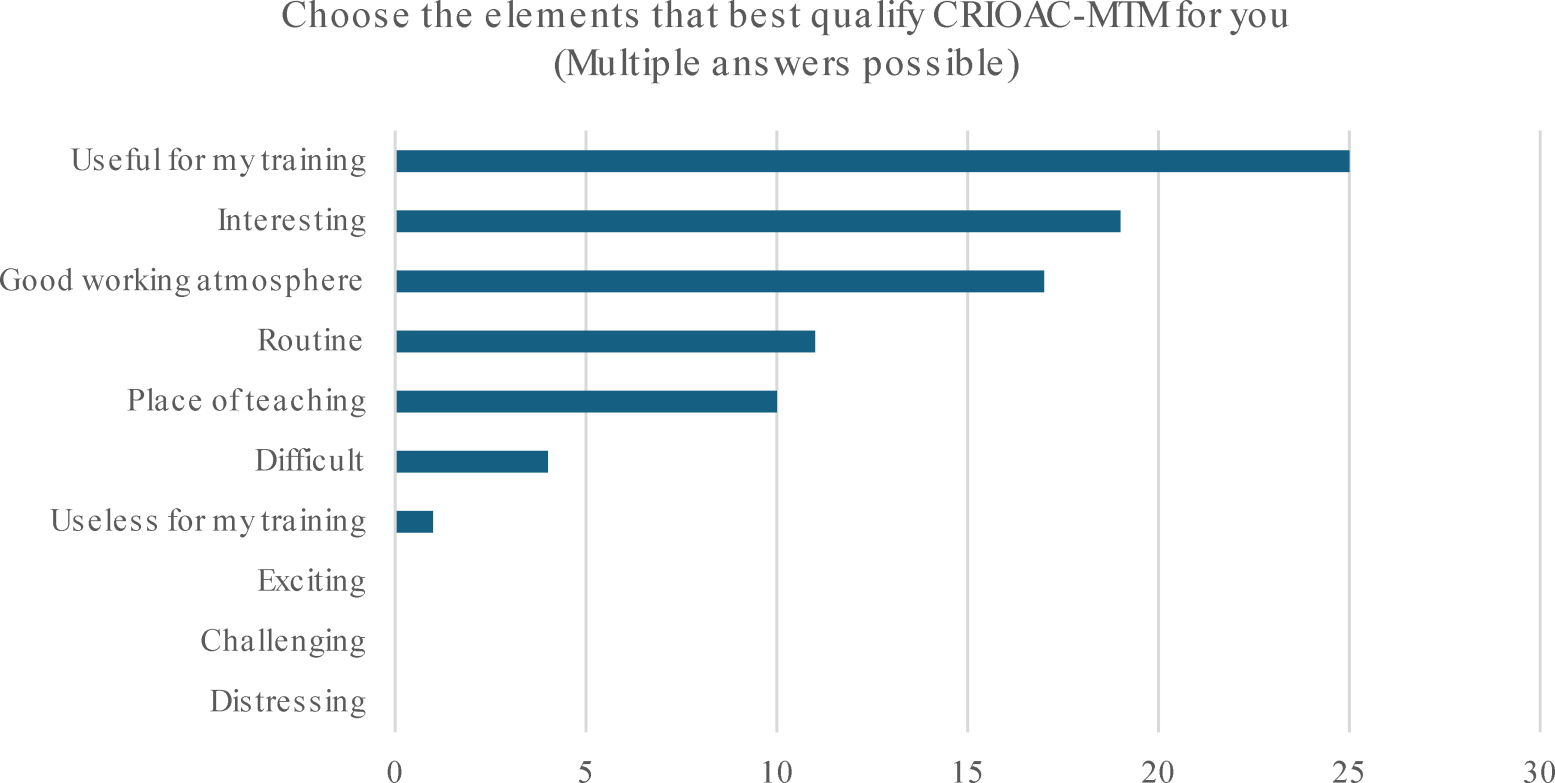
Perception of CRIOAC-MTM by orthopedic residents

**Figure 3:**
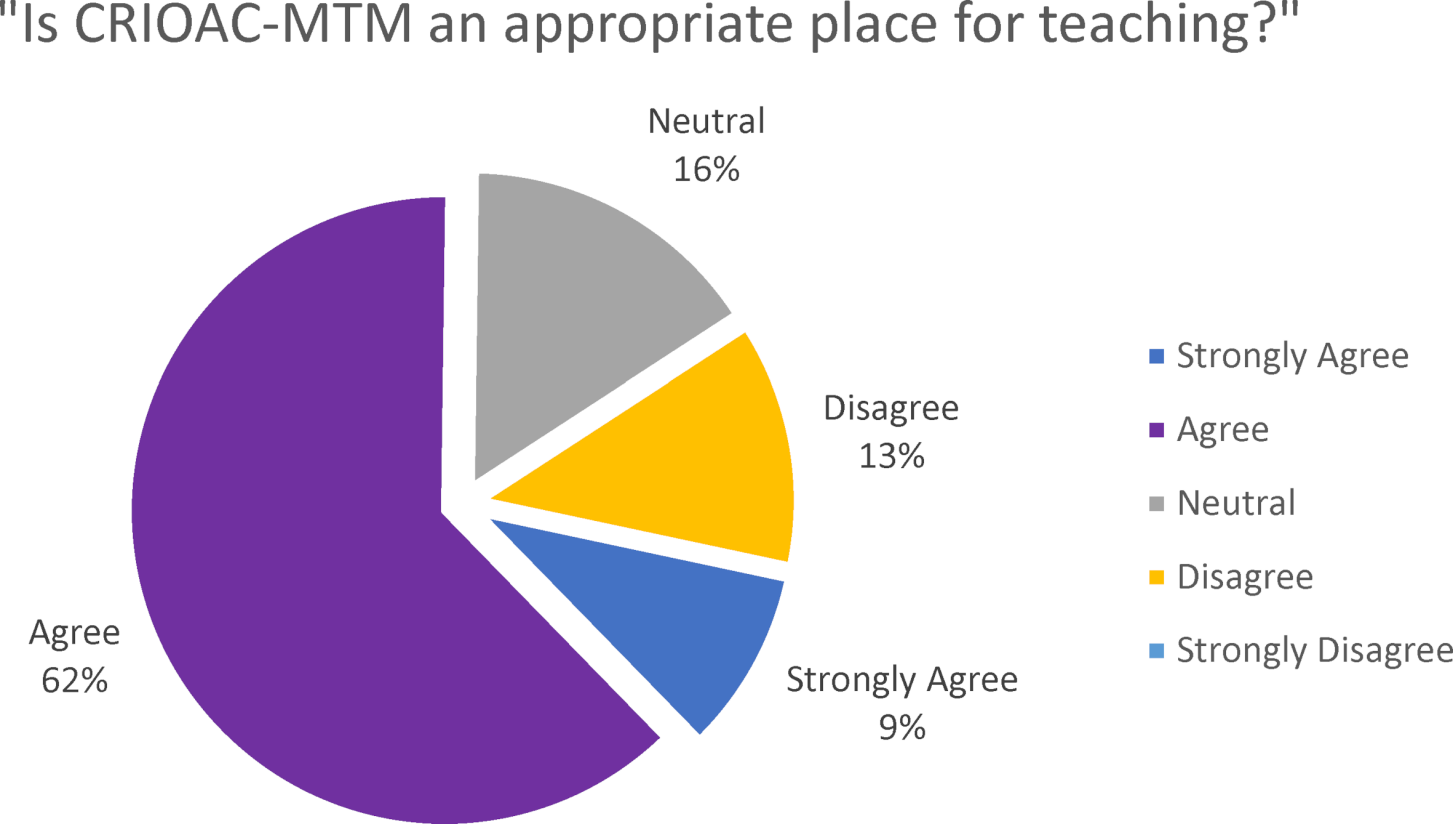
Evaluation of the perception of CRIOAC-MTM as a suitable place for teaching

Regarding the perception of MTM by the orthopedic residents, they were asked to select as many options as applicable among a total of ten options that most accurately depicted their perception of the MTM. These items included distressing, challenging, difficult, routine, useless for my training, useful for my training, interesting, exciting, place of teaching, and good working environment. The top chosen item was “useful for my training,” selected by 25 residents (75%) as demonstrated in figure 2 (Fig. 2). In response to the question, “Is MTM an appropriate place for teaching?” 23 residents (71%) expressed a favorable opinion, as shown in figure 3 (Fig. 3).

**Figure 4:**
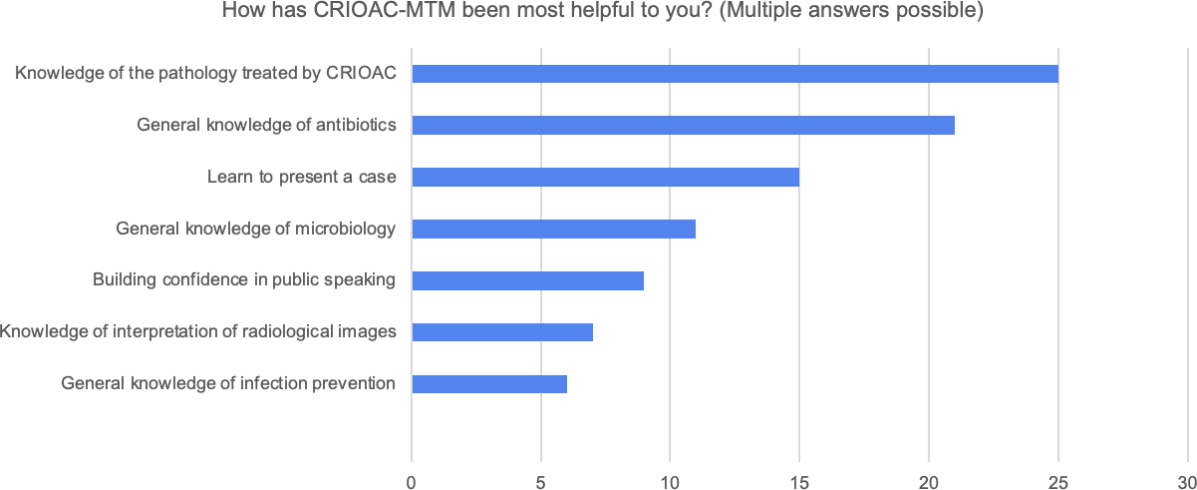
illustrates areas in which CRIOAC-MTM has been most helpful to participants, highlighting key areas of knowledge and skill development

For acquired knowledge, residents were first asked if CRIOAC MTM has helped them acquire skills or knowledge useful for their daily practice. The results showed an overwhelmingly positive response, with 84% (27/32), while no negative responses were present. Secondly, they were asked if they think they have acquired knowledge about osteoarticular infections thanks to CRIOAC MTM, of which 84% (27/32) answered positively. Then the orthopedic residents were provided with seven items and asked to choose as many as applicable in terms of their perceived benefits. Responses were pooled, and each item was ranked based on the most chosen out of all total responses. Among the completed responses, the highest rank was given to “knowledge of the pathology treated by CRIOAC (complex osteoarticular infections): diagnosis and treatment,” selected by 78% (25/32) of the participants as demonstrated in figure 4 (Fig. 4). When asked about the impact of MTM on their knowledge of the various specialties involved, 84% (27/32) of the respondents responded positively. Additionally, the majority, 78% (25/32) of the orthopedic residents, expressed that their participation in MTM would make it easier for them to access case presentations on osteoarticular infections, if necessary, in their future practice.

Concerning ways of improvement proposed by orthopedic residents, 75% (24/32) expressed their belief that there is potential for improving the teaching during MTM. Residents provided insightful and detailed suggestions to enhance the teaching during CRIOAC MTM. They emphasize being more actively involved in the learning process and suggested asking questions like, “What do you think of the situation?” and “What is your proposed therapy?” based on their level of expertise. This approach is designed to ensure that they do not feel overwhelmed and “humiliated” in front of other specialties. As one resident put it, “Tailoring questions to the resident’s level would make them more comfortable participating.”

**Figure 5:**
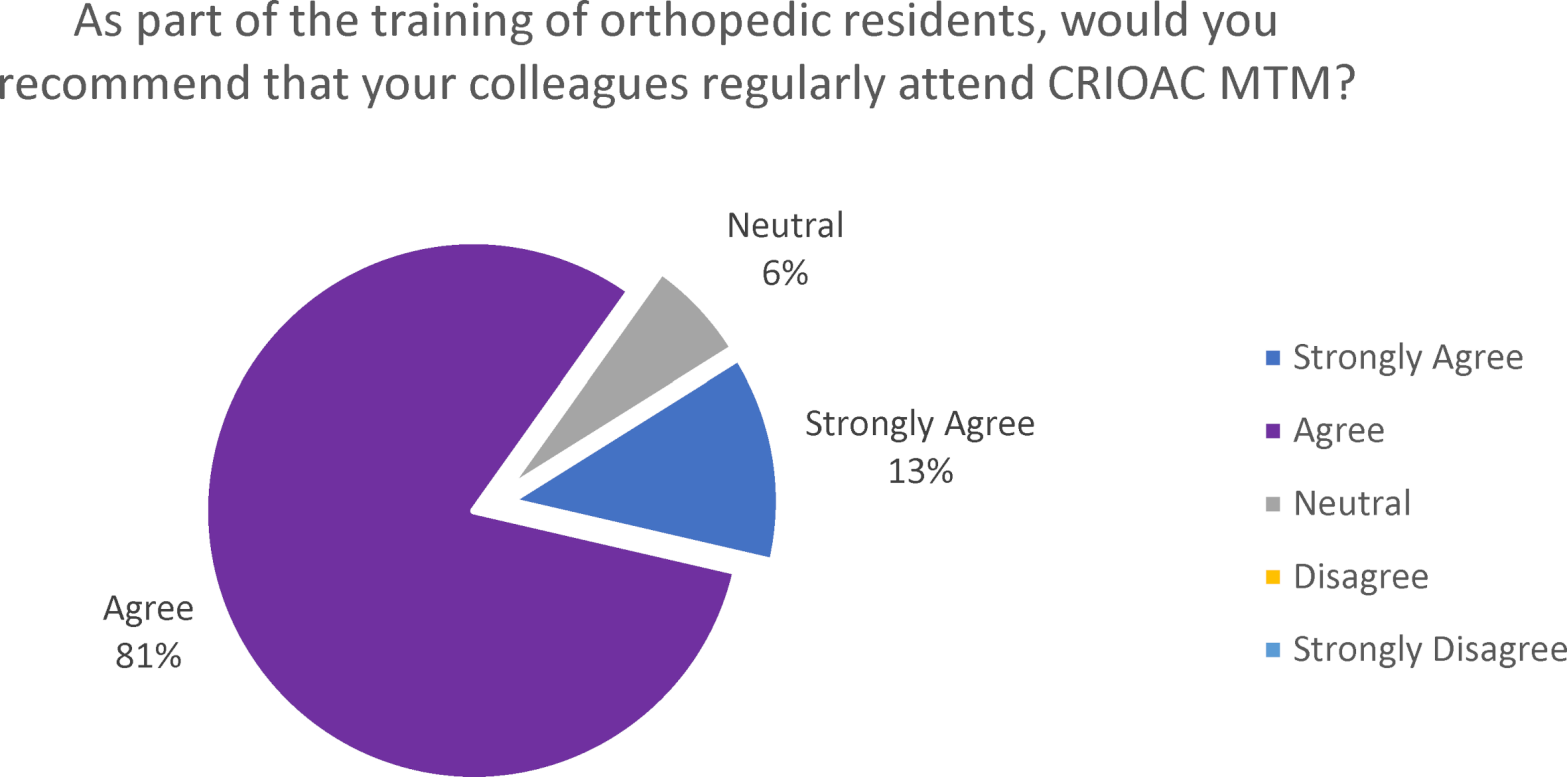
Recommendation for orthopedic residents to attend the CRIOAC-MTM

Regarding recommendations, most orthopedic residents (94%, 30/32) responded positively to the question, “As part of the training of orthopedic residents, would you recommend that your colleagues regularly attend CRIOAC MTM?” as shown in figure 5 (Fig. 5).

Residents also recommend integrating additional classes into the curriculum. They believed that early semester classes focusing on clinical scenarios and antibiotic usage are essential for a solid foundation. According to one resident, “Starting the semester with these classes would help us grasp complex situations better.” Coupled with a variety of learning resources, including classes on antibiotic resistance, antibiotic spectra, and pathogens, these can help residents have a better grasp and confidence with their decision-making on this subject. They also recommend the use of a website for supplemental information as well as creating explanatory decision trees to aid in decision-making. Residents called for more explanation on imaging and the rationale behind antibiotic selection, believing this would improve their understanding of patient care. A resident commented, “Explaining why we choose certain antibiotics and how imaging fits in is essential.”

Residents stressed the importance of taking the time after each case to summarize the patient’s problem comprehensively while highlighting the importance of emphasizing surgical management modalities. They believed this would provide a holistic view of patient care. Some residents found it interesting to propose treatment plans and antibiotics before presenting the official CRIOAC management. When new recommendations are made, residents suggested providing articles as guidance so they can study the recommendations in a more structured manner.

## Discussion

In medical education, the importance of fostering collaboration and shared knowledge among diverse specialties cannot be overstated [11]. The multidisciplinary osteoarticular infection meetings serve as invaluable platforms for teaching, offering a myriad of benefits for residents eager to enhance their knowledge and broaden their perspectives. The results of our study shed light on the perceptions of orthopedic residents regarding CRIOAC-MTMs. The majority viewed it as useful, interesting, and a conducive working environment. 

This perception unveils intriguing and potentially transformative insights. It’s important to acknowledge that MTMs were not explicitly designed for teaching purposes [5]. Yet our research has shown their educational potential. Despite the absence of existing evidence in the medical literature regarding this topic, our findings, drawn from a single MTM, provide a compelling glimpse into the learning opportunities it offers. These intriguing findings prompt us to explore the role of MTMs as a platform for teaching and learning.

This touches back to the similar previously above-mentioned study discussing the same questions in the context of multidisciplinary tumor board meetings. When the two are compared we can see that, Musculoskeletal Tumor Board Meetings (MTBM) and CRIOAC MTMs reveal distinct yet complementary approaches to enhancing medical education through multidisciplinary collaboration. The focus of the MTBM program is orthopedic oncology, providing residents with exposure to a wide range of specialties, such as radiology, oncology, and pathology. Clinical skills are enhanced, referral decisions are refined, and interdisciplinary teamwork is cultivated through this exposure. On the other hand, CRIOAC MTMs are focused exclusively on osteoarticular infections, further developing antibiotic knowledge, imaging modalities, and surgical interventions tailored to complex bone and joint infections. Through these comparative insights, we highlight the variety of educational opportunities offered by multidisciplinary meetings across a range of medical specialties, through which we can refine medical education practices and facilitate interdisciplinary collaboration all of which contribute to well-rounded clinical practice.

Orthopedic residents participating in MTMs have the opportunity to engage with authentic cases, enhancing their clinical reasoning and critical thinking skills. The exposure to a diverse array of complex cases fosters an environment conducive to deep learning and problem-solving.

The positive impact extended beyond orthopedic specialties, with residents recognizing the value in broadening their understanding of various medical specialties involved in the management of complex osteoarticular infections. This interdisciplinary aspect enhances the residents’ holistic approach to patient care. Case-based discussions for example, help residents actively apply theoretical frameworks to complex clinical scenarios, refining their problem-solving and diagnostic skills [11], [12]. While exposure to the collective expertise of various specialties provides a rich context for decision-making, enabling residents to make well-informed choices based on sound clinical judgement. This interdisciplinary collaboration between multiple specialties offers diverse medical perspectives to broaden residents’ understanding of osteoarticular infections. Residents develop effective communication and collaboration skills essential for seamless integration within interprofessional healthcare teams. Orthopedic residents, through their engagement in MTMs, not only acquire medical knowledge but also refine their interpersonal skills and professionalism through effective communication, active participation, and respect for the expertise of other specialties [13]. 

Furthermore, MTMs offer a unique opportunity to teach and reinforce the importance of mutual respect among different medical specialties. The collaborative nature of these meetings fosters an environment where all participants, regardless of their background, can contribute meaningfully to patient care discussions. This not only enhances the learning experience but also reinforces the principles of interdisciplinary teamwork and cooperation [14].

Residents’ feedback emphasizes the importance of tailoring the teaching method to engage the residents effectively. This includes providing multi-faceted learning resources and fostering clear communication and didactic interactions. Their insights highlight the need to actively engage residents by tailoring questions to their level of expertise, ensuring a supportive learning environment, and integrating early semester classes to lay a strong foundation. Additionally, residents stress the importance of diverse learning resources, including lectures on antibiotics, common adverse effects, and decision-making algorithm like explanatory trees for the treatment of each complex BJIs. They advocate for a deeper focus on imaging and antibiotic rationale, along with an emphasis on surgical management. Overall, their recommendations aim to make CRIOAC MTM a more engaging and comprehensive learning experience.

It is also critical to realize the limitations of our study. The review was carried out retrospectively, taking into account changes in survey response durations and the length of residents’ rotations. The survey itself had shortcomings, such as an overreliance on closed-ended questions and only undergoing face validation, even though it had already been published. Furthermore, no objective evaluation of orthopedic residents was performed. 

Nonetheless, these limitations are tolerable considering that the study’s primary goal was to focus on residents’ impressions of learning opportunities during the MTM, a previously unexplored subject. 

## Conclusion

Our study provides a glimpse into the multifaceted potential of MTMs in medical education, extending beyond their primary function in patient care. These meetings offer grounds for teaching, enriching the clinical judgment, professionalism, and collaborative skills of orthopedic residents. The interprofessional nature of MTMs allows for case-based discussions that transcend disciplinary boundaries, promoting a holistic approach to patient care. While further research is warranted to fully explore the educational benefits of MTMs, our findings underscore the intriguing possibilities they hold for enhancing medical training and fostering collaborative healthcare practices.

## Notes

### Institutional review board statement

The study was conducted in accordance with the Declaration of Helsinki, and approved by the Institutional Review (project N° 2023 050)

### Informed consent statement

Informed consent was obtained from all participants involved in the study.

### Authors’ contributions

RS, OA: Study design and conceptualization, writing and revising the manuscript. JB, LRLN, ML: data collection and curation, review and editing of the manuscript. All authors have read and approved the final version of the manuscript.

### Authors’ ORCIDs

- Ramy Samargandi: [0000-0001-5912-9667]
- Osamah Abualross: [0009-0000-2750-9876]
- Marion Lacasse: [0000-0001-7372-6361]
- Louis-Romée Le Nail: [0000-0003-1642-4935]
- Julien Berhouet: [0000-0001-6718-261X]

## Acknowledgements

The authors thank the orthopedic residents participating in the study. The authors also express their sincere gratitude to Susanne Junghans for her invaluable assistance with the proofreading of the German text.

## Competing interests

The authors declare that they have no competing interests.
